# Muscone and (+)-Borneol Cooperatively Strengthen CREB Induction of Claudin 5 in IL-1*β*-Induced Endothelium Injury

**DOI:** 10.3390/antiox11081455

**Published:** 2022-07-26

**Authors:** Yu-Chen Li, Yi Li, Yu-Ning Zhang, Qiong Zhao, Pei-Lin Zhang, Meng-Ru Sun, Bao-Lin Liu, Hua Yang, Ping Li

**Affiliations:** State Key Laboratory of Natural Medicines, China Pharmaceutical University, No. 24 Tongjia Lane, Nanjing 210009, China; 3219020396@stu.cpu.edu.cn (Y.-C.L.); 3221020587@stu.cpu.edu.cn (Y.-N.Z.); 3120020088@stu.cpu.edu.cn (Q.Z.); 3220020273@stu.cpu.edu.cn (P.-L.Z.); 3220020408@stu.cpu.edu.cn (M.-R.S.); 1019870913@cpu.edu.cn (B.-L.L.); yanghua@cpu.edu.cn (H.Y.)

**Keywords:** muscone, (+)-borneol, CREB, claudin 5, cerebral microvascular endothelium

## Abstract

Claudin 5 is one of the major proteins of tight junctions and is responsible for cerebrovascular integrity and BBB function. Muscone and (+)-borneol is the major ingredient of moschus and borneolum, respectively, with antioxidative and anti-inflammatory activities. This study investigated whether muscone and (+)-borneol combination protected claudin 5 by targeting ROS-mediated IL-1*β* accumulation. Muscone and (+)-borneol reduced cerebral infarct volume and cerebrovascular leakage with claudin 5 protection in mice after stroke, largely due to inhibiting ROS accumulation and inflammatory infiltrate of microglia. Muscone reduced ROS and then blocked the CaN/Erk1/2 pathway to decrease IL-1*β* release, while (+)-borneol removed mitochondrial ROS and attenuated the SDH/Hif-1*α* pathway to inhibit IL-1*β* transcription, thereby jointly reducing IL-1*β* production. Accumulated IL-1*β* disrupted cAMP/CREB activation and attenuated transcriptional regulation of claudin 5. Muscone and (+)-borneol combination cooperatively protected BBB function by blocking IL-1*β*-mediated cAMP/CREB/claudin 5 cascades. Mutation of Ser133 site of CREB or knockdown of claudin 5 weakened the effects of muscone and (+)-borneol on upregulation of TEER value and downregulation of FITC-dextran permeability, suggesting that targeting CREB/claudin 5 was an important strategy to protect vascular integrity. This study provided ideas for the studies of synergistic protection against ischemic brain injury about the active ingredients of traditional Chinese medicines (TCMs).

## 1. Introduction

The blood–brain barrier (BBB) is a multicellular vascular structure that is composed of tightly bound cerebral endothelial cells, pericytes, and perivascular astrocytes, and its integrity is essential for neuroprotection from peripheral risk factors [1]. As a unique feature of endothelial cells, tight junctions (TJs) contribute to sealing barriers for BBB integrity [2]. Claudins are one of the major proteins of TJs, and they bind to scaffolding proteins such as zonula occludens-1/2/3 (ZO-1/2/3) and then indirectly link to the actin cytoskeleton via a PDZ-binding motif at the C-terminal [3]. Loss of claudins is associated with BBB breakdown in neurodegenerative diseases, such as Huntington’s disease (HD) and Alzheimer’s disease (AD) [4], as well as acute brain injury including ischemic stroke and depression [5,6]. Claudin 5 is regarded as the most important claudin to maintain TJs because it is mostly enriched in the endothelial layer of the brain [7]. Mice with claudin 5 deficiency have a BBB permeability to small molecules, resulting in death within 10 h after birth [7]. Based on this, claudin 5 has been considered a potential target for BBB protection during ischemic injury.

Although ischemia occurs locally in the affected brain area, it triggers peripheral stress responses, which drive oxidative and inflammatory damage to the microvascular endothelium and increase BBB permeability [8]. In stressed cells, endotoxin or viral infection triggers oxidative stress to accumulate reactive oxygen species (ROS) and destroy the redox homeostasis, which induces the maturation and release of inflammatory factors and triggers inflammatory reactions. Particulate-matter exposure significantly enhanced the airway inflammatory response through ROS-mediated activation of MAPK and downstream NF-*κ*B signaling pathways [9]. Mitochondrial ROS governed the proinflammatory response by regulating MAPK and NF-*κ*B pathways in microglia [10]. There is evidence indicating that rising inflammatory cytokines in serum with ischemic stroke [11,12] and IL-1*β* is known as the pivotal factor of BBB dysfunction, due to vascular leakage and inflammatory injury [13,14]. IL-1*β* also increases intestinal permeability by degrading occludin in mice with colitis [15]. In rat brain endothelial cells, IL-1*β* induced relocation of ZO-1 and occludin from the plasma membrane to the cytosolic compartment, resulting in BBB permeability disruption [16]. Hence, protection of claudin 5 from oxidation stress-mediated inflammation might be a feasible means to explore potential targets for ischemic stroke.

The Angong Niuhuang Pill (ANP) is a well-known classical prescription of traditional Chinese medicine (TCM), widely used for the management of acute cerebrovascular diseases such as ischemia stroke, intracerebral hemorrhage, and traumatic brain damage [17,18], having the role to prevent neural apoptosis and neurological deficits [19]. Meanwhile, its protective effects on tight junction proteins have been documented [20]. Importantly, the herbal pair of moschus-borneolum is regarded not only as a basic unit of ANP but also a vital component in Chinese traditional prescriptions [21], making an irreplaceable contribution to the therapeutic effect of ANP on cerebrovascular diseases. The compatibility of moschus-borneolum is traditionally viewed as an effective means in clinical application. Notably, a study reported the neuroprotective effect of moschus compatible with borneolum on ischemia stroke [22]. Muscone is a potent antioxidative and anti-inflammatory agent in moschus [23], while (+)-borneol is a natural bicyclic monoterpene rich in borneolum with significant inhibitory effects on oxidative damage and inflammatory response [24]. Chemical structures of muscone and (+)-borneol are shown in Figure S1. Furthermore, muscone ameliorated lipopolysaccharide (LPS)-induced depressive-like behaviors by repressing neuroinflammation in the prefrontal cortex of mice [25]. (+)-Borneol maintained the integrity of BBB via protecting TJs expression during ischemic injury [26]. However, the effects of combined administration of muscone and (+)-borneol on ischemic brain injury have not been clarified.

Consistent with their effects on oxidation and inflammation inhibition and cerebral protection, in the present study, we further demonstrated that combined administration of muscone and (+)-borneol synergically protected claudin 5 stability and cerebral microvascular integrity by eliminating ROS accumulation and inhibiting IL-1*β* secretion in cerebral microvascular endothelial cells. ROS removed by muscone attenuated calcium overload-mediated Erk1/2 inflammatory pathway, while the scavenging effect of (+)-borneol on mitochondrial ROS inhibited SDH enzyme activity and blocked succinate/HIF-1*α* inflammatory pathway. (+)-Borneol played an anti-inflammatory role in terms of metabolic regulation, which was different from muscone. Hereafter, their roles focused on inhibiting IL-1*β* maturation and secretion, jointly enhancing claudin 5 expression, and protecting BBB function by activating the cAMP/CREB cascade. This study provided scientific data for revealing the pharmacological effect of a moschus-borneolum drug pair on improving cerebrovascular diseases and expanding its clinical application and provided a certain reference for further studying the synergistic effects of various active ingredients in TCM compounds.

## 2. Method

### 2.1. Chemicals and Reagents

Muscone and (+)-borneol (purity ≥ 98%) were purchased from Chengdu Must Biotechnology Co., Ltd. (Chengdu, China). Rose bengal (330000), 2,3,5-triphenyl tetrazolium chloride (TTC, T8877), Evans blue (E2129), lipopolysaccharide (LPS, L2880), dimethyl malonate (DMM, 136441), dimethyl succinate (W239607) and *N*-acetyl-L-cysteine (NAC, A9165) were purchased from Sigma (St. Louis, MO, USA), Cyclosporin A (CSA, HY-B0579), calcimycin (HY-N6687) and mito-TEMPO (HY-112879) were obtained from Med Chem Express (Brea, CA, USA). IL-1*β* (AF-211-11B) was purchased from Peprotech (Cranbury, NJ, USA).

### 2.2. Animals and Treatment

All animal experiments were approved by the Animal Ethics Committee of China Pharmaceutical University (protocol code: 2021-12-006 and date of approval: December 2021) following the National Institutes of Health and ARRIVE (Animal Research: Reporting of In Vivo Experiments) guidelines. Male C57BL/6J mice (six to eight-week-old, 20–22 g) were purchased from the Comparative Medical Center of Yangzhou University (Yangzhou, China). Animals were housed in a standard environment with constant temperature and humidity and a 12 h light–dark cycle. The mice were given a standard diet and water ad libitum.

Cortical microvascular photothrombosis was employed for photothrombotic stroke (PT) in mice as previously described [27]. Briefly, mice were anesthetized and then placed in a stereotactic frame (Stoelting, Wood Dale, IL, USA). After incising the midline of the skin to expose the skull, a cold light source (11,500 lux) with a 2 mm diameter was fixed on 1.5 mm to the right of the bregma point. Animals then received Rose Bengal solution (Sigma; 100 mg/kg) intraperitoneally and after 5 min the brain was illuminated for 15 min. The scalp was sutured and the mice were allowed to recover on a heating pad. Mice in the sham group were injected with the same dose of Rose Bengal without illumination. In this study, mice were arbitrarily and equally assigned into 5 groups: sham group, model group, model + muscone (2 mg/kg, i.g.) group, model + (+)-borneol (40 mg/kg, i.g.) group, model + muscone (2 mg/kg, i.g.) + (+)-borneol (40 mg/kg, i.g.) group. After photothrombotic stroke surgery, mice were given intragastric administration once a day for 3 consecutive days. Two hours after the last administration, the infarct volume and Evans Blue extravasation in the brain were measured. Meanwhile, brain tissues were harvested and fixed for immunofluorescence detection, and blood was collected for the analysis of inflammatory factors.

For the establishment of middle cerebral artery occlusion (MCAO), male C57BL/6J mice (weight 20–22 g) were anesthetized with isoflurane 3% for induction and 1.5% for maintenance (in a mixture of 30% oxygen and 70% nitrous oxide). Briefly, the carotid artery, external carotid artery (ECA), and internal carotid artery (ICA) were exposed, and a silicon-coated filament was inserted into ICA from ECA to occlude the MCA. After 1 h of ischemia, reperfusion was performed by removing the filament. To access whether the animals treated with MCAO had been successfully modeled, cerebral blood flow (CBF) was monitored via a laser speckle flow imaging system (FLPI2, Moor) before MCAO, after MCAO and reperfusion (Figure S2). Before 30 min of occlusion and 4 h after occlusion, the MCAO mice were given muscone (2 mg/kg, i.g.), (+)-borneol (40 mg/kg), or combined administration of muscone and (+)-borneol. Mice in the sham and MCAO groups were given an equal volume of normal saline.

After 24 h reperfusion, the neurological deficit of the mice was accessed according to the Zea-Longa [28]: 0 suggests no obvious deficit; 1 suggests a failure to stretch contralateral forelimb while tail pulled; 2 suggests spontaneous contralateral turning; 3 suggests spontaneous contralateral circling; 4 suggests loss of walking ability.

### 2.3. 2,3,5-Triphenyl-Tetrazolium Chloride (TTC) and Evans Blue Staining

Infarct volume was analyzed with TTC staining after photothrombotic stroke and middle cerebral artery occlusion (MCAO) surgery. After the mice were euthanized, the brains were rapidly isolated and frozen, and then the tissue was sectioned into 2 mm-thick coronal slices. Subsequently, these brain slices were stained with 1% TTC in a 37 °C water bath for 5–10 min and then fixed in 4% formaldehyde overnight. The unstained infarct area was quantified with Image J software 1.52a (NIH, Washington, USA).

BBB integrity was determined by the extravasation of Evans blue (EB) after photothrombotic stroke. Briefly, EB solution (2%, 4 mL/kg) was injected into the tail vein and circulated for 2 h. Subsequently, the mice were anesthetized and transcardially perfused with 0.9% saline and 4% paraformaldehyde sequentially. Finally, brains were collected and photographed.

### 2.4. Cell Culture

bEnd.3 cells were purchased from iCell Bioscience Inc (Shanghai, China) and incubated in Dulbecco’s modified eagle medium (DMEM, KeyGEN BioTECH, Nanjing, China) with 10% (*v*/*v*) fetal bovine serum (FBS, Gibco, New York, NY, USA). All cells were cultured at 37 °C in the 5% CO_2_ humidified incubator.

### 2.5. Transfection of bEnd.3 Cells

The bEnd.3 cells at 70–80% confluence were transfected with *Creb* siRNA, *Cldn5* siRNA, or negative control (NC) to specifically suppress *Creb* or *Cldn5* expression with Lipofectamine™ 3000 transfection reagent (Invitrogen™, L3000015, Carlsbad, CA, USA). The siRNA sequences were shown below. *Creb* siRNA: 5′-CGUAGAAAGAAGAAAGAAUTT-3′; *Cldn5* siRNA: 5′-CUGCUAACCUGA AAGGGCA-3′. NC siRNA: 5′-UUC UCCGAA CGUGUC ACGU-3′. For CREB overexpression or CREB mutation, bEnd.3 cells were transfected with pcDNA3.1(+)-M_*Creb*, pcDNA3.1(+)-M_*Creb*(p.S133R)-HA (p.S133S→R) plasmids, or pc DNA3.1(+)-M_NC plasmids (Genomeditech Co., Ltd., Shanghai, China). After 48 h of transfection, cells were treated with a fresh medium for further experiments.

### 2.6. Transendothelial Electrical Resistance (TEER) Measurement and FITC-Dextran Paracellular Permeability Determination

TEER value is commonly used to assess endothelial barrier function in vitro. bEnd.3 cells suspension was seeded in the upper chamber of 12-well Transwell inserts (3460, CORNING, New York, NY, USA) at a volume of 600 μL/well, and 1.5 mL of DMEM medium was added to the lower chamber. The cells were incubated at 37 °C and 5% CO_2_ for 7 days until a confluent monolayer of cells was formed. After treating the cells with the indicated reagents for 16 h, the chambers were cleaned with Hanks’ Balanced Salt Solution (HBSS), and then TEER was measured with an R/V Meter of Epithelium (RE1600, Beijing KingTech Technology Co. Ltd., Beijing, China). The TEER values of the cell layers were obtained by subtracting the resistance of background TEER values in the cell-free insert and then multiplied by the total membrane surface area to obtain the resistance value in Ω·cm^2^.

For evaluating the paracellular permeability, 500 μL of DMEM containing 1 mg/mL FITC-Dextran (70 kDa, Sigma, St. Louis, MO, USA) was added to the upper chamber. After incubating at 37 °C for 1 h from light, the fluorescence intensity of FITC-Dextran transferred to the lower chamber was measured using a multimode microplate reader (BERTHOLD Technologies, Bad Wildbad, Germany) with excitation at 485 nm and emission at 530 nm. The permeability coefficient was calculated using the following equation: P_dextran_ = (RFU_lower_/RFU_upper_) (V) (1/time) (1/area), and normalized to control untreated cultures.

### 2.7. Human Interactome and Pathway-Enrichment Analysis

The human interactome used in this study contains 332,749 pairwise binding interactions between 18,508 human proteins [29], assembled from 21 public databases that compile experimentally derived protein–protein interactions (PPI) data: (1) binary PPIs, derived from high-throughput yeast two-hybrid (Y2H) experiments and three-dimensional protein structures; (2) PPIs identified by affinity purification followed by mass spectrometry (AP-MS); (3) kinase-substrate pairs identified by literature-derived low-throughput or high-throughput experiments; (4) signaling interactions; (5) regulatory interactions. Kyoto Encyclopedia of Genes and Genomes (KEGG) enrichment analyses were performed to evaluate the functional pathways regulated by IL-1*β* using cluster Profiler.

### 2.8. Quantitative Real-Time PCR (qRT-PCR)

RNA isolator (Vazyme, Nanjing, China) was used following the manufacturer’s protocol to extract the total RNA from bEnd.3 cells. The purity and concentration of isolated RNA were determined with a NANO-100 microspectrophotometer (ALLSHENG, Hangzhou, China), and an equal amount of RNA was reverse-transcribed into cDNA using an HiScript^®^ Q RT SuperMix for qPCR (Vazyme, Nanjing, China). Real-time PCR was then performed on LightCycler 480 II (Roche, Switzerland) with the primers and ChamQ SYBR Color qPCR Master Mix (Vazyme, Nanjing, China). The mRNA levels were normalized against *Actb* and quantified using the 2^−ΔΔCt^ method. Primer sequences are listed in Table S1.

### 2.9. Western Blotting

The total protein of bEnd.3 cells was lysed with RIPA buffer containing phosphatase and protease inhibitors (Roche, Switzerland). Nuclear extracts were prepared using a Nuclear and Cytoplasmic Protein Extraction Kit (Beyotime, P0028, Shanghai, China) following the manufacturer’s protocol. The concentration of protein was quantified using BCA protein assay kit (Beyotime, P0011, Shanghai, China). Equal amounts of protein samples were loaded on a 10% SDS-PAGE gel and then transferred to nitrocellulose filter (NC) membranes after being electrophoresed. Membranes were blocked by immersion in 5% non-fat milk for 2 h and then immunoblotted with primary antibody (anti-p-ERK1/2, 1:1000, Bioworld Technology Cat# BS4621, RRID:AB_1663587; anti-ERK1/2, 1:1000, Bioworld Technology Cat# BS3628, RRID:AB_1662299; anti-p-p38 MAPK, 1:1000, Cell Signaling Technology Cat# 9215, RRID:AB_331762; anti-p38, 1:1000, Bioworld Technology Cat# BS3566, RRID:AB_1663652; anti-p-SAPK/JNK (Thr183/Tyr185), 1:1000, Cell Signaling Technology Cat# 4668, RRID:AB_823588; anti-JNK1/2/3, 1:1000, Bioworld Technology Cat# BS1544, RRID:AB_1664025; anti-NLRP3, 1:1000, Abcam Cat# ab270449; anti-p-NF-*κ*B p65 (Ser536), 1:1000, Cell Signaling Technology Cat# 3033, RRID:AB_331284; anti-NF-*κ*B p65, 1:1000, Santa Cruz Biotechnology Cat# sc-8008, RRID:AB_628017; anti-TLR4, 1:1000, Santa Cruz Biotechnology Cat# sc-293072, RRID:AB_10611320; anti-Hif-1*α*, 1:1000, Cell Signaling Technology Cat# 36169, RRID: AB_2799095; anti-p-PKA C, 1:1000, Cell Signaling Technology Cat# 5661, RRID:AB_10707163; anti-PKA C, 1:1000, Cell Signaling Technology Cat# 5842, RRID:AB_10706172; anti-claudin 5, 1:1000, Bioworld Technology Cat# BS1069, RRID: AB_1664057; anti-p-CREB (Ser 133), 1:1000, Cell Signaling Technology Cat# 9198, RRID:AB_2561044; anti-CREB, 1:1000, Cell Signaling Technology Cat#9197, RRID:AB_331673; anti-ZO-1, 1:1000, Abcam Cat# ab216880, RRID:AB_2909434; anti-occludin, 1:1000, Bioworld Technology Cat# BS72035; *β*-actin, 1:2000, Proteintech Cat# 20536-1-AP, RRID: AB_10700003; anti-Histone H3, 1:1000, Cell Signaling Technology Cat#9715; RRID:AB_331563) overnight at 4 °C. After three washes with Tris-buffered saline-Tween (TBST) buffer, strips were incubated with goat anti-rabbit IgG (H + L) HRP (1:10,000, Bioworld Technology Cat# BS13278, RRID: AB_2773728) or Goat Anti-Mouse IgG (H + L) HRP, (1:10,000, Bioworld Technology Cat# BS12478, RRID: AB_2773727) at room temperature for 1 h. Finally, the signal was visualized by an ECL kit (Tanon, 180-5001, Shanghai, China) and the data were analyzed by Image-Pro Plus 6.0 (IPP 6.0) software (Media Cybernetics, Silver Spring, USA).

### 2.10. Immunofluorescence Staining

For detection of the protein expressions of CD31, claudin 5, and Iba1 in brain tissue of the mice treated with photothrombotic stroke, the brain tissue was fixed and embedded in Tissue-Tek O.C.T. Compound (Sakura Finetek, Tokyo, Japan), and then cut into 5 μm-thick slides. The slides fixed with 4% paraformaldehyde were permeabilized with 0.3% Triton X-100 and then blocked with goat serum. After that, the slides were incubated with primary antibodies (anti-CD31, 1 μg/mL, Abcam Cat# ab9498, RRID: AB_307284; anti-claudin 5, 1:200, Bioworld Technology Cat# BS1069, RRID: AB_1664057; anti-Iba1, 1:800, Abcam Cat# ab178847, RRID: AB_2832244) overnight at 4 °C, followed by incubation with second antibody (Goat Anti-Mouse IgG H&L (Alexa Fluor^®^ 647), 1:500, Abcam Cat# ab150115, RRID: AB_2687948; Donkey Anti-Rabbit IgG H&L (Alexa Fluor^®^ 647), 1:500, Abcam Cat# ab150075, RRID: AB_ 2752244) at room temperature for 2 h. After washing with PBS, the slides were stained with DAPI (Bioworld Technology, BD5010, St. Paul, MN, USA) and treated with an antifluorescence quenching agent (Beyotime, P0126, Shanghai, China). Fluorescence images were visualized and quantified by a confocal laser-scanning microscope (Zeiss, LSM 800, Jena, Germany).

For detection of intracellular immunofluorescence, the treated bEnd.3 cells were permeabilized with 0.1% Triton X-100 after fixation in 4% paraformaldehyde. After blocking with 5% bovine serum albumin (BSA), the cells were immunostained with anti-HIF-1*α* anti-HIF-1*α* (1:800, Cell Signaling Technology Cat# 36169, RRID: AB_2799095) or anti-CREB (1:500, Cell Signaling Technology Cat#9197, RRID: AB_331673) at 4 °C overnight, and subsequently incubated with Goat anti-Rabbit IgG H&L (Alexa Fluor^®^ 488) (1:500, Abcam Cat# ab150077, RRID: AB_2630356) for 2 h in darkness. Finally, the nucleus was stained with DAPI (1:1000, Bioworld Technology, BD5010). Fluorescence images were visualized and quantified by a confocal laser-scanning microscope (Zeiss, LSM 800, Jena, Germany).

### 2.11. Measurement of ROS, Mitochondrial ROS, and Intracellular Calcium Contents

To observe ROS production in brain tissue of the mice from photothrombotic stroke and bEnd.3 cells, the tissue slices, and treated bEnd.3 cells were stained with 10 μM DCFH-DA (Beyotime, S0033S) and DAPI (Bioworld Technology, BD5010) at 37 °C for 20 min protected from light. Images of brain slices were viewed with the confocal laser-scanning microscope (Zeiss, LSM 800, Jena, Germany).

To observe the accumulation of mitochondrial ROS, the treated bEnd.3 cells were incubated with 5 μM MitoSOX™ red mitochondrial superoxide indicator (Invitrogen™, M36008, Carlsbad, CA, USA) reagent at 37 °C for 10 min, followed by 50 nM Mito-tracker green (Beyotime, C1048, Shanghai, China) reagent for 30 min in the dark. The nucleus was visualized by incubating with Hoechst (1:1000, Invitrogen™, H3570) for 10 min. Images were acquired and quantified by a confocal scanning microscope (Zeiss, LSM 800, Jena, Germany).

For the detection of intracellular calcium content, bEnd.3 cells with indicated treatment were incubated with Fluo-8AM (1 μg/mL, Abcam, ab142773, Cambridge, UK) for 30 min. After washing with PBS, the relative fluorescence intensity was detected using a multimode microplate reader (BERTHOLD Technologies, Bad Wildbad, Germany) at excitation of 490 nm and emission of 520 nm.

### 2.12. Dual-Luciferase Reporter Assay

The Mouse_*Cldn5* promoter (starting from1978 bp upstream (position-1978) to 1 bp upstream (position-1) of the TSS) was synthesized and inserted in the pGL3-basic vector between Mlul and Xhol sites (Genomeditech Co., Ltd., Shanghai, China). *Creb* plasmids were transfected into bEnd.3 cells together with pcDNA3.1-M_*Creb* plasmids or pcDNA3.1(+)-M_NC plasmids with Lipofectamine™ 3000 transfection reagent (Invitrogen™, L3000015, Carlsbad, CA, USA) for 48 h. Cells were lysed with passive lysis buffer (Promega, E1941, Madison, WI, USA) and then the luciferase activities were measured by the dual-luciferase assay system (Promega, E2920, Madison, WI, USA).

### 2.13. Chromatin Immunoprecipitation (ChIP)

ChIP analysis was carried out using SimpleChIP Enzymatic Chromatin IP Kit (Cell Signaling, 9002, Danvers, MA, USA) following the manufacturer’s guidelines. Briefly, the treated bEnd.3 cells were cross-linked with 1% paraformaldehyde for 10 min at room temperature and then blocked with glycine. Chromatin was harvested using micrococcal nuclease digestion and sheared by sonication. The sheared chromatin was divided into two parts, one of which served as input control. The remaining portion was incubated with the primary antibody of CREB (1:50, Cell Signaling Technology Cat# 9197, RRID: AB_331673), at 4 °C overnight and then the complexes were captured by protein A/G plus agarose beads, with IgG as a negative control. After washing with ChIP elution buffer, the immunoprecipitated complex was treated with protease to release DNA fragments. Subsequently, DNA was purified with columns and amplified by quantitative polymerase chain reaction (qPCR) with site-specific primers. Primer sequences are listed in Table S2.

### 2.14. Biochemical Assay

The blood samples collected from the experimental animal were allowed to stand and centrifuged at 1000× *g* for 15 min to obtain serum. The mouse IL-1*β* and IL-6 ELISA Kit (CUSABIO, Wuhan, China) were used to determine the levels of IL-1*β* and IL-6, respectively.

After treating bEnd.3 cells with the corresponding drugs, the supernatant was collected for the measurement of IL-1*β* (CUSABIO, Wuhan, China). In addition, the treated bEnd.3 cells were washed with PBS and collected with cell lysis buffer. After centrifuging at 12,000× *g* for 15 min at 4 °C, the supernatant was collected for the measurement of sAC, cAMP, and CaMKII according to the protocol of sAC ELISA Kit, cAMP ELISA Kit (Elabscience, Wuhan, China) and CaMKII ELISA Kit (Meimian, Yancheng, China). The quantification of succinate was measured by succinate colorimetric assay kit (Sigma, MAK184, St. Louis, MO, USA) The activity of CaN and SDH were detected according to the calcineurin assay kit (Jiancheng, A068-1-1, Nanjing, China) and succinate dehydrogenase assay kit (Jiancheng, A022-1-1, Nanjing, China).

### 2.15. Drug–Drug Synergy Determination

To determine drug synergy for inhibiting ROS and IL-1*β* production, bEnd.3 cells were plated in 96-well and 48-well plates, respectively. bEnd.3 cells were treated with muscone and (+)-borneol, which was serial twofold diluted in each step in the presence of LPS, resulting in four concentrations for each ingredient, combining a matrix of 25 different concentration ratios for both ingredients. After 16 h, bEnd.3 in 96-wells were detected ROS production by a microplate reader (BERTHOLD Technologies, Bad Wildbad, Germany). The supernate of bEnd.3 cells in a 48-well plate was used for measuring the IL-1*β* production.

### 2.16. Statistical Analysis

All statistical analyses were performed using GraphPad Prism 8.0 software. The data were presented as means ± SD (*n* = 5). All experiments were randomized and blinded. After being calculated for normal distribution with the Shapiro–Wilk test, statistical comparisons between different groups were evaluated by one-way ANOVA followed by Tukey’s test (>two groups) or *t*-test (two groups). Values of *p* < 0.05 were considered statistically significant.

## 3. Results

### 3.1. Combined Administration of Muscone and (+)-Borneol Protects Brain Microvessels against Ischemic Brain Injury

**Figure 1:**
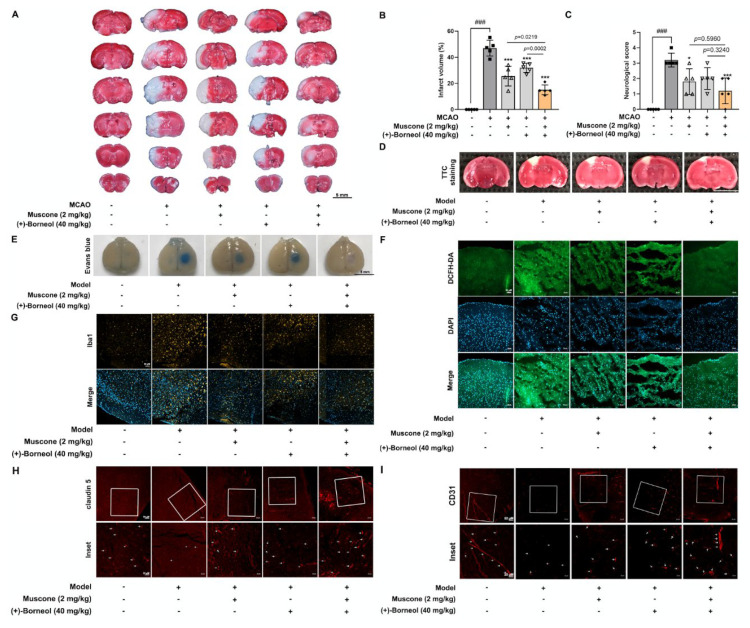
Combined administration of muscone and (+)-borneol protects brain microvessels and against ischemic brain injury. (**A**–**C**): The MCAO model was established after 1 h ischemia and 24 h reperfusion in C57BL/6J mice. Muscone (2 mg/kg), (+)-borneol 40 mg/kg, or muscone combined with (+)-borneol were given intragastric administration 30 min before ischemia and 4 h after ischemia, respectively. (**A**,**B**) cerebral infarction area was assessed by TTC staining and calculated by Image J with the following equation: the infarct volume (%) = (right hemisphere − non-infarct of the left hemisphere)/right hemisphere size × 100% (*n* = 5, scale bar: 5 mm); (**C**): neurological score was evaluated (*n* = 5). (**D**–**I**): Mice were treated with intragastric administration of muscone and (+)-borneol 30 min before the photothrombotic stroke, followed by continuous administration for 3 d. (**D**) cerebral infarct area was assessed by TTC staining (*n* = 5, scale bar: 5 mm); (**E**) mice were treated with 2% Evans blue for 2 h and then perfused with normal saline and 4% paraformaldehyde. Brain tissue was removed and photographed (*n* = 5, scale bar: 5 mm); (**F**): the ROS production in brain peri-infarct zones was determined by DCFH-DA staining (*n* = 5, scale bar: 50 μm); (**G**) immunofluorescence staining of Iba1 for activated microglia in brain peri-infarct zones (*n* = 5, scale bar: 50 μm). (**H**) microvascular endothelium labeled by claudin 5 and (**I**) CD31 in peri-infarct regions were observed by immunofluorescence (*n* = 5, scale bar: 50 μm or 20 μm). Data are presented as mean ± SD (*n* = 5). * *p* < 0.05, *** *p* < 0.001 vs. MCAO; ^###^ *p* < 0.001 vs. indicated treatment. *p* values are determined by one-way ANOVA followed by Tukey’s test.

Given its traditional application in ischemic stroke, we established the MCAO mouse model and photothrombotic stroke model in male C57BL/6J mice that were sensitive to brain ischemic injury [30], and the mice were given 2 mg/kg muscone and 40 mg/kg (+)-borneol by intragastric administration, and the doses were chosen based on the previous studies [31,32]. Both muscone and (+)-borneol reduced the volume of cerebral infarction and improved the neurobehavior in MCAO mice. Combined administration of muscone and (+)-borneol significantly enhanced the protective effects (Figure 1A–C). Furthermore, in the photothrombotic stroke model, we observed more effective effects after muscone and (+)-borneol treatment in combination, as evidenced by the significant reduction in infarct volume and the Evans blue extravasation into the brain parenchyma (Figure 1D,E). The green fluorescence of DCFH-DA in the peripheral infarct area was significantly enhanced, indicating the occurrence of oxidative stress, whereas muscone and (+)-borneol significantly reduced ROS accumulation, and the combined administration of the two drugs had a stronger effect on antioxidant damage (Figure 1F). Meanwhile, muscone and (+)-borneol reduced inflammatory cell recruitment in the peri-infarct zones in a similar way (Figure 1G). We continued to investigate the regulation of tight junction protein in ischemic injury and observed that muscone and (+)-borneol upregulated the expression of claudin 5 and CD31 in the peri-infarct zones, and more potent effects were shown in combinatory treatment (Figure 1H,I). The results demonstrated that combinatory administration of muscone and (+)-borneol protected brain microvessels against ischemic injury via inhibiting oxidation damage and inflammatory response.

### 3.2. Muscone and (+)-Borneol Protect Endothelial Function with Inhibition of IL-1β Production

**Figure 2:**
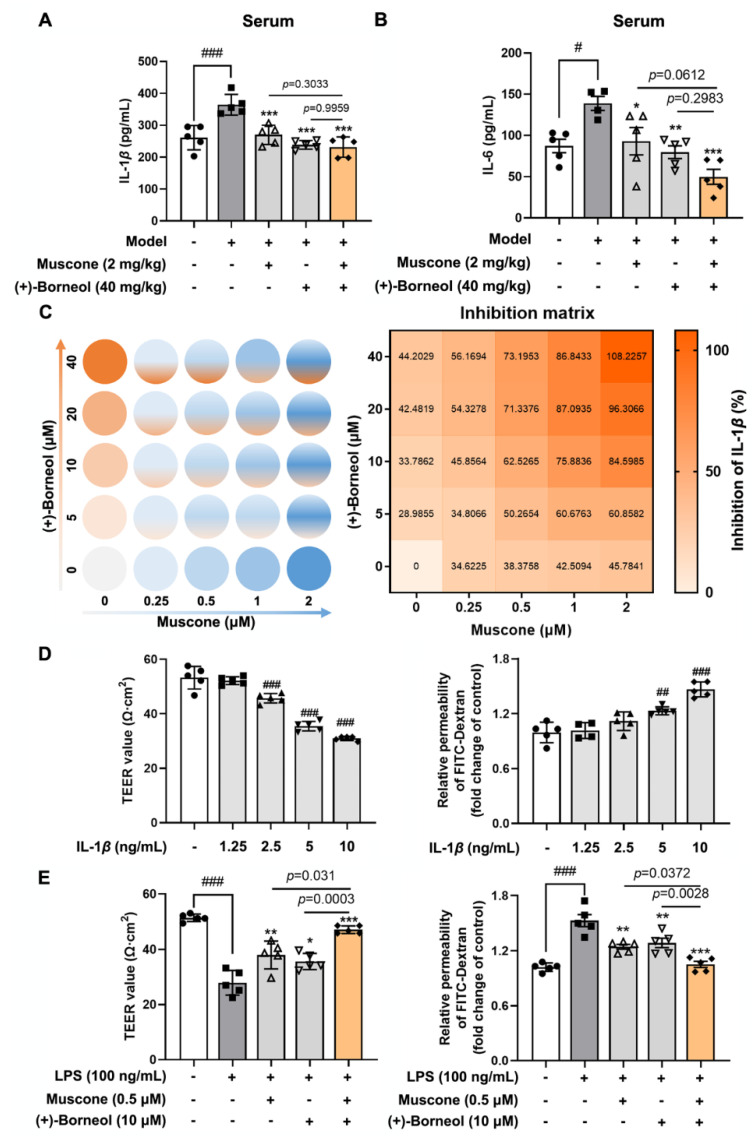
Muscone and (+)-borneol protect endothelial function by reducing IL-1*β* release. (**A**,**B**) the levels of IL-1*β* and IL-6 in the serum of mice treated with photothrombotic stroke were detected by Elisa kit (*n* = 5); (**C**) the inhibition matrix of IL-1*β* production in LPS-induced bEnd.3 cells treated with muscone (dose range from 0 to 2 μM) and (+)-borneol (dose range from 0 to 40 μM) for 16 h; (**D**,**E**) relative transepithelial electrical resistance (TEER) value and FITC-dextran (70 kDa) permeability of bEnd.3 monolayers cultured in a 0.4 μm Transwell membrane were measured when exposed to IL-1*β* insult. Data are presented as mean ± SD (*n* = 5). * *p* < 0.05, ** *p* < 0.01, *** *p* < 0.001 vs. Model or LPS treatment; ^#^ *p* < 0.05, ^##^ *p* < 0.01, ^###^ *p* < 0.001 vs. indicated treatment or untreated group. *p* values are determined by one-way ANOVA followed by Tukey’s test.

Brain ischemia induced peripheral inflammatory responses, as expressed by an elevated level of proinflammatory cytokines in the blood. Muscone and (+)-borneol reduced IL-1*β* and IL-6 production, but no potent effect was observed after combined administration, possibly because the inhibition effects of each treatment alone had reached the baseline (Figure 2A,B). Next, we assessed the efficiency of muscone and (+)-borneol treatment in combination using a 5 × 5 combination matrix, in which the two agents were titrated using a twofold dilution in each step, and quantified the resulting IL-1*β* inhibition parameters in LPS-induced bEnd.3 cells, which is a cell line derived from murine endothelioma widely used for the study of brain microvascular endothelial cells. No impact on cell survival was observed when incubated with muscone at concentrations ranging from 1 μM to 8 μM, while (+)-borneol was added up to 640 μM (Figure S3A,B). As shown in Figure 2C, the inhibition effect on IL-1*β* production was enhanced with increasing dosages of muscone and (+)-borneol, and combinatorial effects were stronger than individual effects. IL-1*β* disrupted vascular integrity through the plasmalemma receptor IL-1R1 activation in different cells, including the endothelial cells [33]. Thus, we performed in vitro endothelial integrity assays using TEER value and FITC-dextran (70 kDa) permeability and found that IL-1*β* stimulation decreased the TEER values and increased the paracellular diffusion of FITC-dextran across endothelial monolayer in a dosage-dependent manner (Figure 2D). LPS stimulation showed similar destruction to IL-1*β* on vascular integrity, whereas muscone and (+)-borneol reversed these changes, and more potent effects were observed in combined treatment (Figure 2E). These results indicated that muscone and (+)-borneol cooperated to combat inflammation and protect endothelial integrity and function.

### 3.3. IL-1β Impairs cAMP/CREB Cascades to Reduce Claudin 5 Expression in Cerebral Microvascular Endothelium

**Figure 3:**
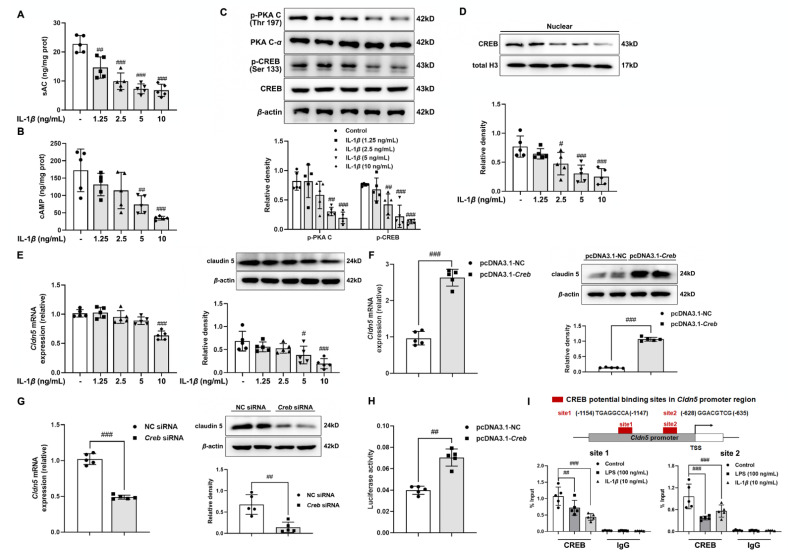
IL-1*β* impairs cAMP/CREB cascades to reduce claudin 5 expression in cerebral microvascular endothelium. (**A**) sAC and (**B**) cAMP contents were detected in bEnd.3 cells when treated with IL-1*β* at the dosage from 1.25 to 10 ng/mL; (**C**) protein expressions of p-PKA C, PKA c-*α*, and p-CREB (Ser 133) in IL-1*β*-stimulated bEnd.3 cells; (**D**) protein expression of CREB in the nucleus of IL-1*β*-stimulated bEnd.3 cells; (**E**) gene and protein expression of claudin 5 in bEnd.3 cells when exposed to IL-1*β* insult; (**F**) gene and protein expression of claudin 5 in bEnd.3 cells transfected with a plasmid for CREB overexpression (*n* = 5); (**G**) gene and protein expression of claudin 5 in bEnd.3 cells transfected with *Creb* siRNA or NC siRNA (*n* = 5); (**H**) bEnd.3 cells were transfected with pcDNA3.1-M_*Creb* plasmids, and then the activity of claudin 5 promotors in endothelial cells was measured (*n* = 5); (**I**) ChIP-quantitative PCR analyses of CREB binding to *Cldn5* promoter in bEnd.3 cells when treated with LPS or IL-1*β* stimulation (*n* = 5). Data are presented as mean ± SD (*n* = 5). ^#^ *p* < 0.05, ^##^ *p* < 0.01, ^###^ *p* < 0.001 vs. untreated group or indicated treatment. *p* values are determined by *t*-test or one-way ANOVA followed by Tukey’s test.

Sepsis is shown to impair CREB in lung endothelial cells, partly due to IL-1*β*-induced vascular leakage [34]. Indeed, the IL-1*β* challenge decreased the activity of soluble adenylyl cyclase (sAC) and the formation of cAMP in endothelial cells (Figure 3A,B). As a consequence, IL-1*β* also inactivated protein kinase A (PKA) and CREB by dephosphorylation in a dosage-dependent manner (Figure 3C), well explaining the inhibition of CREB nuclear transport (Figure 3D). Furthermore, IL-1*β* reduced the gene and protein levels of claudin 5 (Figure 3E). Corresponding changes in protein levels of CREB and claudin 5 were seen when exposed to IL-1*β* insult, implying the relation between CREB transcription and claudin 5 induction. We transfected bEnd.3 cells with a plasmid for CREB overexpression and observed a significant increase in both gene and protein levels of claudin 5 (Figure 3F). In contrast, depletion of CREB by siRNA decreased claudin 5 expression in gene and protein levels, confirming the transcription of CREB on claudin 5 (Figure 3G). Ulteriorly, luciferase reporter assay also showed that forced expression of CREB increased the activity of *Cldn5* promotor in microvascular endothelial cells (Figure 3H), indicative of the induction of claudin 5 by CREB transcription. CREB is known to bind preferentially with a canonical CRE sequence 5′-TGACGTCA-3′. JASPAR database predicted two potential CREB-binding sites in the *Cldn5* promoter region, which displayed significant homology with the canonical CREB recognition motif. Chromatin immunoprecipitation (ChIP)-PCR assay showed that LPS and IL-1*β* challenge attenuated the binding of CREB to the promoter region of *Cldn5* (Figure 3I), thereby reducing the expression of claudin 5 to destroy the tight junctions. These results raised the possibility that IL-1*β* impaired CREB-mediated claudin 5 transcription to induce endothelial dysfunction.

### 3.4. Muscone and (+)-Borneol Strengthen cAMP/CREB/Claudin 5 Signaling to Maintain Cerebral Microvascular Integrity

**Figure 4:**
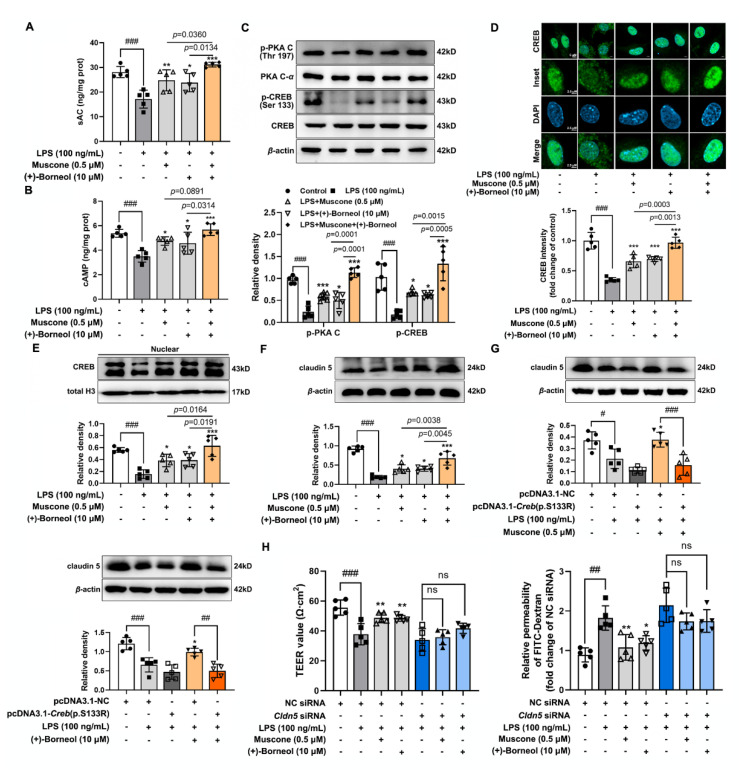
Muscone and (+)-borneol strengthen cAMP/CREB/claudin 5 signaling to maintain cerebral microvascular integrity. (**A**) sAC and (**B**) cAMP contents in the lysis of bEnd.3 cells were detected (*n* = 5); (**C**) protein levels of p-PKA C, PKA c-*α*, p-CREB (Ser 133), and CREB were measured in LPS-stimulated bEnd.3 cells; (**D**) the nuclear translocation of CREB in bEnd.3 cells were labeled by immunofluorescent staining (*n* = 5, scale bar: 5 μm or 2.5 μm); (**E**) protein expression of CREB in the nucleus of LPS-stimulated bEnd.3 cells (*n* = 5); (**F**) protein expression of claudin 5 in bEnd.3 cells treated with muscone and (+)-borneol in the presence of LPS for 16 h; (**G**) protein expression of claudin 5 in bEnd.3 cells when transfected with CREB mutation at Ser133 site and then treated with muscone or (+)-borneol in the presence of LPS (*n* = 5); (**H**) bEnd.3 cells cultured in a 0.4 μm Transwell membrane were transfected with *Cldn5* siRNA. Then, the TEER values and FITC-dextran permeability were measured in the presence of LPS (*n* = 5). Data are presented as mean ± SD (*n* = 5). * *p* < 0.05, ** *p* < 0.01, *** *p* < 0.001 vs. LPS treatment; ^#^ *p* < 0.05, ^##^ *p* < 0.01, ^###^ *p* < 0.001 vs. indicated treatment; ns: no significant different. *p* values are determined by one-way ANOVA followed by Tukey’s test.

Similar to IL-1*β* stimulation, LPS inactivated sAC and reduced cAMP generation in bEnd.3 cells, which were normalized by muscone and (+)-borneol treatment (Figure 4A,B). Consistently, muscone and (+)-borneol increased PKA and CREB activity through phosphorylation modification (Figure 4C) and increased CREB nuclear transport (Figure 4D,E) with claudin 5 induction (Figure 4F). More potent effects were observed when cells were exposed to muscone and (+)-borneol treatment in combination (Figure 4A–F). To investigate the pivotal role of CREB in protecting claudin 5 stability from muscone and (+)-borneol, we mutated CREB Ser 133 to Ala, blocking the phosphorylation of the residue and inactivating CREB. As shown in Figure 4G, LPS stimulation was more destructive to claudin 5, and the enhanced claudin 5 by muscone and (+)-borneol was significantly reduced, supporting that CREB phosphorylation is necessary for the action of muscone and (+)-borneol (Figure 4G). In addition, we investigated the regulatory effect of muscone and (+)-borneol on other important tight junction proteins, such as ZO-1 and occludin, and found that LPS stimulation reduced the protein levels of ZO-1 and occludin, while muscone and (+)-borneol reversed these alterations, and the combined administration of muscone and (+)-borneol had a stronger protective effect (Figure S5). These results suggested that muscone and (+)-borneol had beneficial effects on maintaining tight junction protein stability and BBB function. Next, we continued to investigate the importance of claudin 5 expression in protecting cerebral microvascular integrity and function of muscone and (+)-borneol and found that the beneficial effects of muscone and (+)-borneol on upregulated TEER values and downregulated FITC-dextran (70 kDa) permeability were abolished by claudin 5 knockdown using siRNA in LPS-stimulated endothelial cells (Figure 4H). These results further supported the claim that muscone and (+)-borneol protected endothelial integrity and barrier function, and regulation of cAMP/CREB/claudin 5 signaling cascades against inflammation was a way that underlies their actions.

### 3.5. Muscone Suppresses Oxidation Stress-Mediated Inflammation via Blocking the Ca^2+^/Erk1/2 Signaling Pathway

**Figure 5:**
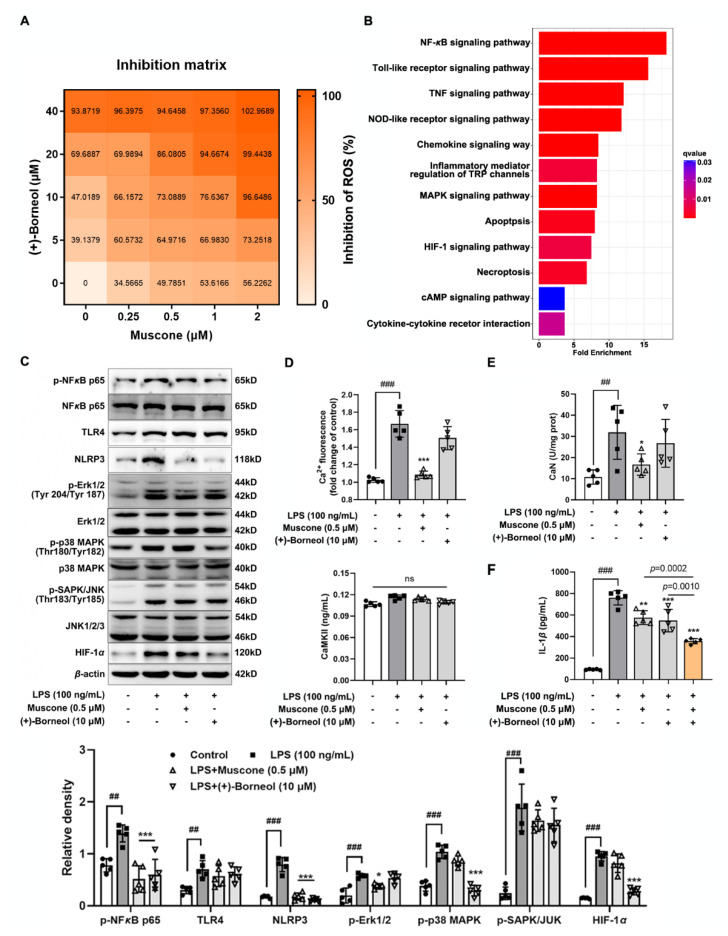
Muscone suppresses oxidation-stress-mediated inflammation via blocking Ca^2+^/Erk1/2 signaling pathway. (**A**) The inhibition matrix of ROS production in LPS-induced bEnd.3 cells treated with muscone (dose range from 0 to 2 μM) and (+)-borneol (dose range from 0 to 40 μM) for 16 h; (**B**) column chart of KEGG pathway enrichment analysis of IL-1*β*-related genes pulled from the human interactome; (**C**) p-NF-*κ*B p65, NF-*κ*B p65, TLR4, NLRP3, p-Erk1/2, Erk 1/2, p-p38 MAPK, p38-MAPK, p-SAPK/JNK, JNK1/2/3, and HIF-1*α* protein expression were determined in LPS-induced bEnd.3 cells (*n* = 5); (**D**) relative fluorescent density of intracellular Ca^2+^ indicated by Fluo 8-AM staining was measured by multimode microplate reader (*n* = 5); (**E**) calcineurin (CaN) activity and Ca^2+^/calmodulin-dependent protein kinase II (CAMKII) activity were measured in LPS-stimulated bEnd.3 cells (*n* = 5); (**F**) IL-1*β* level in bEnd.3 cells when exposed to LPS stimulation (*n* = 5). Data are presented as mean ± SD (*n* = 5). * *p* < 0.05, ** *p* < 0.01, *** *p* < 0.001 vs. LPS treatment; ^##^ *p* < 0.01, ^###^ *p* < 0.001 vs. indicated treatment; ns: no significant difference. *p* values are determined by one-way ANOVA followed by Tukey’s test.

Oxidative damage often occurs with inflammation, and it is the pathological basis of inflammatory response. Given the structural characteristics of muscone and (+)-borneol, as well as their antioxidative and anti-inflammatory activities, we continued to investigate the regulatory effects of muscone and (+)-borneol on ROS clearance. As shown in Figure 5A, the inhibition effect on ROS production was enhanced with increasing dosages of muscone and (+)-borneol, and combinatorial effects were stronger than individual effects. Next, we continued to explore the regulatory role of cleared ROS in the activation of inflammatory pathways, as well as the different effects of muscone and (+)-borneol in this process. We predicted the reported pathways of IL-1*β* production pulled from the human interactome [29,35]. Pathway enrichment analysis showed that five signaling pathways with significant differences closely related to endothelial cell injury were selected for verification, including the NF-*κ*B signaling pathway, Toll-like receptor signaling pathway, NOD-like receptor signaling pathway, MAPK signaling pathway, and HIF-1 signaling pathway (Figure 5B). As shown in Figure 5C, muscone significantly inhibited the expression of p-NF-*κ*B, NLRP3, and p-Erk1/2, while (+)-borneol suppressed the expression of p-NF-*κ*B, NLRP3, p-p38 MAPK, and HIF-1*α* induction (Figure 5C). To distinguish the different pathways of muscone and (+)-borneol inhibiting inflammatory response, we focused the action pathways of muscone on the Erk1/2 pathway. Intracellular calcium disturbance affects downstream signaling cascades that involve MAPKs, including Erk1/2. As expected, LPS stimulation induced calcium overload labeled with Fluo-8 AM probe, whereas muscone significantly reversed this alteration by reducing fluorescence intensity compared to (+)-borneol (Figure 5D). Activation of Erk1/2 pathway is closely related to intracellular Ca^2+^ binding to calmodulin and then activation of Ca^2+^/calmodulin-dependent protein kinase II (CAMKII) or calcineurin (CaN). Indeed, LPS stimulation activated CaN without affecting CaMKII in bEnd.3 cells and muscone reduced upregulation of CaN while borneol had no significant effect (Figure 5E). As a result, muscone reduced the production of IL-1*β* (Figure 5F). These results suggested that blocking the Ca^2+^/Erk1/2 signaling pathway might be a way for muscone to suppress oxidation-stress-mediated inflammation.

### 3.6. (+)-Borneol Suppressed Mitochondrial ROS-Mediated Inflammatory Response by Inhibiting Succinate/Hif-1α Signaling Cascades

**Figure 6:**
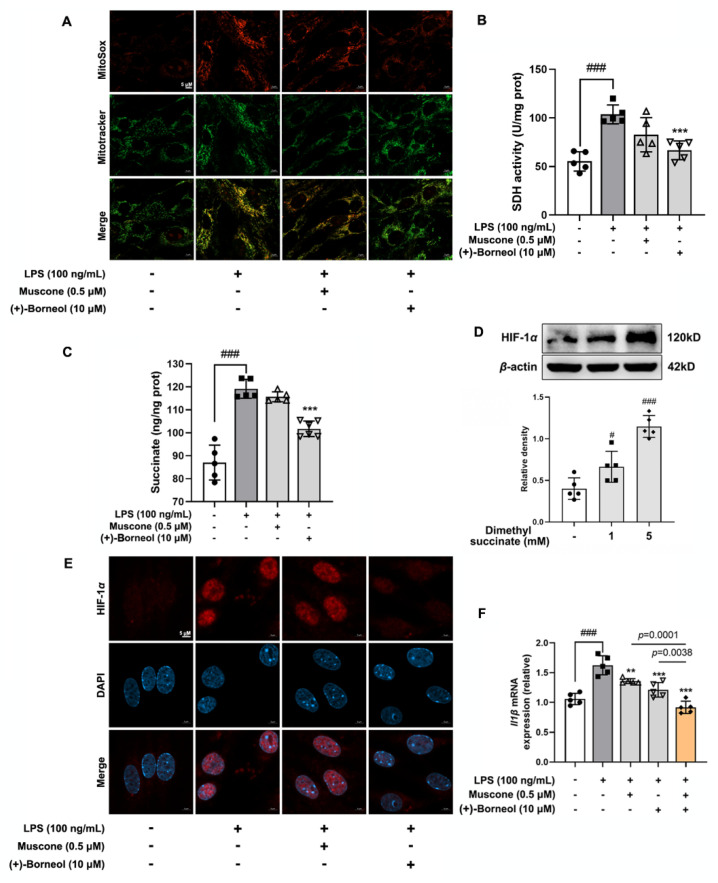
(+)-Borneol suppressed mitochondrial ROS-mediated inflammatory response by inhibiting succinate/Hif-1*α* signaling cascades. (**A**) Mitochondrial ROS production was indicated by MitoSox probe staining (red) (*n* = 5, scale bar: 5 μm); (**B**) succinate content was assessed in LPS-stimulated bEnd.3 cells (*n* = 5); (**C**) succinate dehydrogenase (SDH) activity (*n* = 5); (**D**) protein expression of Hif-1*α* in bEnd.3 cells treated with dimethyl succinate (*n* = 5); (**E**) Hif-1*α* translocation was indicated by immunofluorescence (*n* = 5, scale bar: 5 μm); (**F**) Il1*β* mRNA level of bEnd.3 cells treated with LPS (*n* = 5). Data are presented as mean ± SD (*n* = 5). ** *p* < 0.01, *** *p* < 0.001 vs. LPS treatment; ^#^ *p* < 0.05, ^###^ *p* < 0.001 vs. indicated treatment or untreated group. *p* values are determined by one-way ANOVA followed by Tukey’s test.

By comparing the characteristics of (+)-borneol inhibiting inflammatory responses, we focused on the HIF-1 signaling pathway, which plays an important role in intracellular metabolism regulation as a transcription factor, especially closely related to mitochondrial metabolism. More notably, mitochondria are also the main organelles producing ROS in cells, so we continued to observe the accumulation of mitochondrial ROS and the regulatory role of borneol. (+)-Borneol significantly reduced LPS-induced mitochondrial ROS production in endothelial cells, while the role of muscone was weaker (Figure 6A). Mitochondrial ROS production is caused by an abnormal mitochondrial electron-transport chain, and the reverse activation of SDH is the trigger [36]. In line with this, (+)-borneol significantly reduced SDH activity (Figure 6B), along with a decrease in succinate concentration (Figure 6C), since SDH is a key enzyme that catalyzes the conversion of succinate to fumarate. Mitochondrial succinate entered the cytoplasm through the SLC25A10 channel and increased HIF-1*α* accumulation via prolyl hydroxylase enzyme (PHD) inactivation [37]. In support, membrane-permeable dimethyl succinate concentration-dependently increased HIF-1*α* protein accumulation in endothelial cells (Figure 6D). LPS stimulation also stabilized HIF-1*α* with nucleus translocation, but these alternations were reversed by (+)-borneol treatment (Figure 6E). HIF-1*α* is a transcription factor that controls gene induction encoding IL-1*β* [38]. As expected, (+)-borneol significantly decreased gene expression of *Il1β* (Figure 5F) in LPS-induced bEnd.3 cells. These results suggested that (+)-borneol suppressed mitochondrial ROS-mediated inflammatory response by inhibiting succinate/Hif-1*α* signaling cascades from the perspective of regulating cell metabolism, which is a way different from muscone.

### 3.7. Calcium Overload and Succinate Accumulation Collaboratively Suppress IL-1β/CREB/Claudin 5 Signaling in the Context of Oxidation Stress

**Figure 7:**
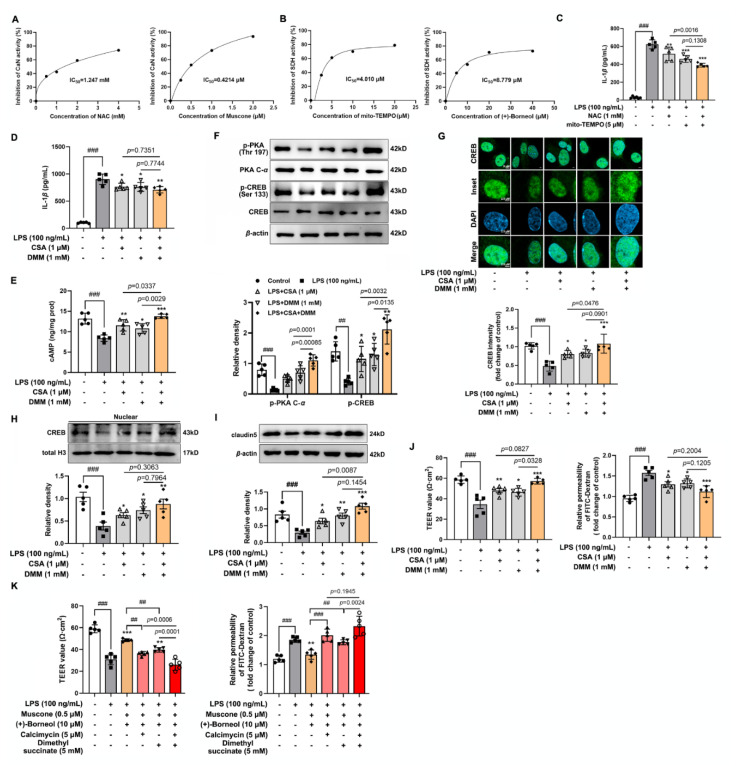
Calcium overload and succinate accumulation collaboratively suppress IL-1*β*/CREB/claudin 5 signaling in the context of oxidation stress. (**A**) bEnd.3 cells were given the indicated dosage of NAC or muscone. The activity of CaN was detected and IC_50_ value was calculated; (**B**) bEnd.3 cells were given the indicated dosage of Mito-TEMPO or (+)-borneol. SDH activity was detected and the IC_50_ value was calculated; (**C**,**D**) the level of IL-1*β* released in LPS-induced bEnd.3 cells with the indicated reagents; (**E**) cAMP level in the lysis of bEnd.3 cells when exposed to LPS stimulation (*n* = 5); (**F**) protein levels of p-PKA C, PKA c-*α*, p-CREB (Ser 133), and CREB in LPS-stimulated bEnd.3 cells; (**G**) immunofluorescence staining indicated nuclear translocation of CREB when treated with CSA and DMM in the presence of LPS (*n* = 5, scale bar: 5 μm or 2.5 μm); (**H**) protein expression of CREB in the nucleus of LPS-stimulated bEnd.3 cells (*n* = 5); (**I**) protein expression of claudin 5 in LPS-induced bEnd.3 cells (*n* = 5); (**J**,**K**) the TEER values and the diffusion of FITC-dextran through the bEnd.3 cells were measured with the indicated reagents (*n* = 5). Data are presented as mean ± SD (*n* = 5). * *p* < 0.05, ** *p* < 0.01, *** *p* < 0.001 vs. LPS treatment; ^##^ *p* < 0.01, ^###^ *p* < 0.001 vs. indicated treatment. *p* values are determined by one-way ANOVA followed by Tukey’s test.

To further clarify the regulatory role of muscone and (+)-borneol in blocking the activation of inflammatory pathways by eliminating ROS accumulation, the ROS-specific inhibitor NAC and the mitochondrial ROS inhibitor Mito-TEMPO were selected to investigate their effects on IL-1*β* production. CaN and SDH were the key enzymes of muscone and (+)-borneol to reduce IL-1*β* production, respectively, and the regulation of NAC and mito-TEMPO has been particularly noted. Sure enough, NAC dose-dependently reduced the activity of CaN with an IC_50_ value of about 1.247 mM, similar to the effect of muscone (Figure 7A). Mito-TEMPO also reduced SDH activity in a dose-dependent manner and the IC_50_ was 4.010 μM, similar to the effect of (+)-borneol (Figure 7B). Consistently, NAC and mito-TEMPO significantly reduced IL-1*β* production, similar to CSA, an inhibitor of CaN, and DMM, an inhibitor of SDH (Figure 7C,D). Next, CSA and DMM were used to investigate whether blocking different inflammatory pathways played a cooperative enhancing role in CREB-mediated claudin 5. CSA and DMM restored cAMP production and phosphorylation of PKA and CREB against LPS insult, thereby promoting CREB nuclear transport (Figure 7E–H). Subsequently, the protein expression of claudin 5 was significantly increased by CSA and DMM (Figure 7I). As a result, the endothelial barrier function was preserved by CSA and DMM, as evidenced by the upregulated TEER value and downregulated FITC-dextran permeability in LPS-induced bEnd.3 cells (Figure 7J). Moreover, potent effects were observed after CSA and DMM treatment in combination (Figure 7D–J). Finally, when supplemented with calcimycin (calcium ionophore) and dimethyl succinate, the collaborative protective effect of muscone and (+)-borneol on endothelial integrity was attenuated. Meanwhile, the inhibitory effect was significantly enhanced when calcimycin and dimethyl succinate were added together (Figure 7K). These results provided evidence that muscone and (+)-borneol suppressed ROS-mediated inflammatory responses in different ways, working in cooperation to protect endothelial function.

## 4. Discussion

Traditional Chinese medicine is commonly prescribed in combination form, consisting of various herbs with dozens of active components, likely due to the advantage of solving complex diseases through the integration of multipathway or multitarget manners [39]. Moschus and borneolum have been used as a common drug pair in traditional prescriptions, and the role is shown to ameliorate cerebral diseases dependent on different conditions. Although the synergistic effects on ischemia stroke in rats have been validated in the previous study [22], our study presented that muscone and (+)-borneol worked together to increase claudin 5 expression and resultantly protected BBB integrity against ischemic brain injury in the context of oxidative stress and inflammatory activation. Muscone reduced ROS to block the Erk1/2 inflammatory pathway, while (+)-borneol removed mitochondrial ROS to weaken the inflammatory transcriptional regulation of Hif-1*α*. Combined with their structural characteristics, muscone and (+)-borneol exerted antioxidant and anti-inflammatory effects through different pathways. Moreover, we revealed that CREB transcriptionally regulated claudin 5 induction, and inflammatory cytokine IL-1*β* impaired cAMP/CREB signaling to reduce claudin 5 in endothelial cells, addressing that protection of claudin 5 stability was a way for pharmacological intervention to ensure the integrity of cerebral microvascular.

Cerebral thrombosis is a sequence of endothelial dysfunction devoted to ischemic injury. In line with previous studies of anti-ischemic effects [26,40], muscone and (+)-borneol reduced infarct volume after stroke in mice by protecting BBB integrity and barrier function. In patients who were suffering from acute stroke, the magnitude of inflammatory response was positively correlated with the extent of ischemic injury to a certain extent [11]. Muscone relieved inflammatory pain by inhibiting microglial activation-mediated inflammatory response via inactivation of NOX4 and NLRP3 inflammasome [23]. (+)-Borneol protected oxygen-glucose deprivation/reperfusion-induced cortical neurons via antioxidation and anti-inflammation through the NF-*κ*B signaling pathway [24]. Consistently, in our study, we found that muscone and (+)-borneol reduced cerebral infarct volume and cerebrovascular leakage with claudin 5 protection in mice after stroke, largely due to inhibiting ROS accumulation and inflammatory microglia recruitment in the peripheral infarct area. Due to the superimposed antioxidative and anti-inflammatory pathways of muscone and (+)-borneol, the combined administration of the two drugs significantly enhanced cerebral protection. Thus, we addressed that the suppression of oxidation damage and inflammatory injury should be an important means for the synergistic effects of muscone and (+)-borneol in the protection of brain endothelium.

IL-1*β* acts in a paracrine and autocrine manner through activating IL-1R1 in the endothelium, leading to increased recruitment of inflammatory cells into the brain parenchyma [33]. Consistently, in our study, with the increase in IL-1*β* concentration, the damage to BBB function was aggravated. Previous research confirmed that IL-1*β*-mediated BBB damage largely depends on TJs’ destruction, including phosphorylation, degradation, and relocation in the context of ischemic injury [41]. In the current study, IL-1*β* downregulated the gene and protein level of claudin 5, contributing to cerebral microvessel damage. Muscone and (+)-borneol functioned to protect cerebral microvessels via preserving TJs stability against ischemic injury [26,42]. Owing to the combinative effect on restraining IL-1*β* release, muscone and (+)-borneol mutually enhanced each other to protect claudin 5 expression from inflammatory insult. This suggests that targeting claudin 5 stability to increase the resistance to proinflammatory stimulation was an important strategy for the improvement of BBB function.

CREB is a transcription factor that binds to the cAMP-response element (CRE) to trigger gene transcription [43]. Activated sAC catalyzes the formation of cAMP and then activates PKA to regulate CREB activity by phosphorylation [43]. CREB is activated in the ischemic brain to promote functional recovery via transcriptional regulation of genes encoding cell survival-related molecules, growth factors, and structural proteins [44]. TJ contains a variety of transmembrane proteins, including claudins and occludin, as well as intracellular scaffold proteins such as ZO-1, which play a key role in the regulation of endothelial monolayer resistance, paracellular solute permeability, and BBB function. Intriguingly, studies have shown that CREB binds to transcriptional promoters including ZO-1 and claudins [45,46], which maintains the integrity and function of brain microvascular. Our study was carried out under the background of LPS-stimulated inflammatory response activation to produce IL-1*β* accumulation and accumulated IL-1*β* disrupted cAMP/CREB activation and attenuated transcriptional regulation of CREB. CREB is known to bind preferentially with a canonical CRE sequence 5′-TGACGTCA-3′. JASPAR database predicted two potential CREB-binding sites in the *Cldn5* promoter region, which displayed significant homology with the canonical CREB recognition motif. ChIP-qPCR and luciferase reporter gene analysis confirmed the transcriptional regulation of claudin 5 by CREB. In LPS-stimulated bEnd.3 cells, muscone and (+)-borneol promoted claudin 5 expression through the activated cAMP/CREB pathway. Notably, the combined administration of the two drugs significantly increased the expression of claudin 5 by the up-regulated CREB transcription, which is closely related to the combined antioxidant and anti-inflammatory effects of muscone and (+)-borneol.

Given the important role of inflammatory factors in the destruction of the cerebrovascular barrier, it is particularly important to elucidate the pathway of IL-1*β* production and to trace the action targets of muscone and (+)-borneol. Oxidative damage is closely related to inflammatory response and accumulated ROS activated MAPK and NF-*κ*B pathways to cause airway inflammation [9]. In activated microglia, mitochondrial ROS activated the MAPK pathway to promote inflammatory response [10]. In our study, muscone and (+)-borneol reduced intracellular ROS and mitochondrial ROS accumulation, thereby affecting different inflammatory pathways. Activated Erk1/2 signaling strengthened inflammatory response during cerebral ischemic injury [47]. In LPS-stimulated bEnd.3 cells, muscone inhibited phosphorylation of Erk1/2 by inhibiting CaN activity. Meanwhile, (+)-borneol attenuated the HIF-1*α* signaling pathway by inhibiting SDH activity, which was closely related to reduced mitochondrial ROS. Therefore, we proposed that regulation from different ways should be the reason for muscone and (+)-borneol combination’s potential role in IL-1*β* suppression. However, we should note that the antioxidative and anti-inflammatory effects were not the only means of pharmacological intervention for ischemic brain injury. In the complex central nervous system, neurons, astrocytes, and microglia play a key role in brain homeostasis, inhibiting apoptosis and maintaining physiological functions of cells are also of great significance. Muscone and (+)-borneol, as active ingredients that can easily enter the BBB, may play a synergistic role in maintaining brain tissue homeostasis through multitarget and multi-pathway by acting on different cells.

## 5. Conclusions

In general, we concluded that ROS-accumulation-mediated inflammatory pathway activation underlies microvessel endothelial cell destruction during cerebral infarction, and protection of claudin 5 stability is an important strategy to improve cerebrovascular integrity. The combined administration of muscone and (+)-borneol increased claudin 5 expression to rescue cerebrovascular endothelial cells from endotoxin insult by inhibiting the IL-1*β*/cAMP/CREB signaling cascades, largely due to protecting redox homeostasis. It is suggested that claudin 5 can be used as a potential drug target to protect BBB function and improve brain injury in clinical studies. In addition, other tight junction proteins such as ZO-1 and occludin are also important targets in clinical studies of cerebral infarction. In addition, an Angong Niuhuang pill is often used in the treatment of stroke and cerebral infarction, and the moschus-borneolum herbal pair can easily pass through the blood–brain barrier (BBB) and play an important role in protecting neurons. Our study preliminarily confirmed the protective effects of muscone and (+)-borneol as a group of active components on tight junction protein and BBB at the cellular and animal levels. This finding suggested the potential clinical application of muscone and (+)-borneol combination for cerebrovascular protection and provided ideas for the studies of synergistic protection against ischemic brain injury about the active ingredients of TCMs.

## Data Availability

All of the data are contained within the article and the Supplementary Materials.

## Supplementary Materials

The following supporting information can be downloaded at: https://www.mdpi.com/article/10.3390/antiox11081455/s1, Figure S1: The structure of muscone and (+)-borneol; Figure S2: The local cerebral blood flow of MCAO mice; Figure S3: Cytotoxicity of muscone and (+)-borneol on bEnd.3 cells; Figure S4: Small interfering RNA (siRNA) screening; Figure S5: Muscone and (+)-borneol increased ZO-1 and occludin expression. Table S1: Primer sequences for qRT-PCR; Table S2: Primer sequences for ChIP-qPCR.

## Abbreviations

ANP: Angong Niuhuang Pill; BBB: blood–brain barrier; CaN: calcineurin; CBF: cerebral blood flow; CREB: cAMP responsive element-binding protein; CSA: cyclosporin A; DMM: dimethyl malonate; LPS: lipopolysaccharide; NAC: *N*-acetyl-L-cysteine; ROS: reactive oxygen species; SDH: succinate dehydrogenase; TCM: traditional Chinese medicine; TEER: transendothelial electrical resistance; TJ: tight junction; TLR: Toll-like receptor.
