# Supplementary material for: Muscone and (+)-Borneol Cooperatively Strengthen CREB Induction of Claudin 5 in IL-1β-Induced Endothelium Injury
Source: Antioxidants (Basel). 2022 Jul 26;11(8):1455. doi: 10.3390/antiox11081455 (PMC9394259; doi:10.3390/antiox11081455)
Supplement: Supplementary file 1 [file antioxidants-11-01455-s001.zip › Supplemetary Material-v4.pdf]

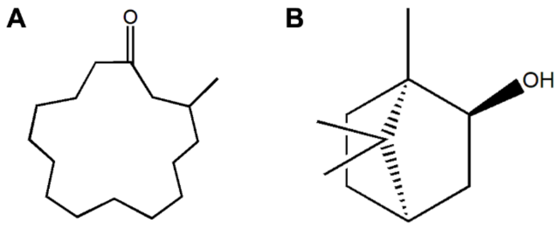

Figure S1 The structure of muscone and (+)-borneol. (A) muscone, (B) (+)-borneol.

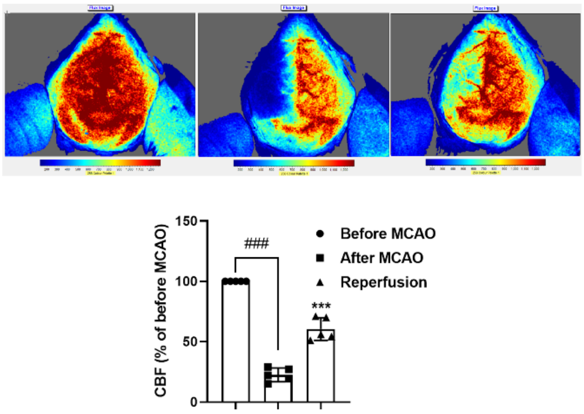

Figure S2 The local cerebral blood flow of MCAO mice. cerebral blood flow before MCAO, after MCAO, and reperfusion (CBF, blood flow in the infarct area/blood flow in the contralateral corresponding area *100%) was reported (n = 5). Data are presented as mean ± SD (n = 5). $^{***}p < 0.001$ vs. after MCAO; $^{\#\#\#}p < 0.001$ *vs.* indicated group. p values are determined by one-way ANOVA followed by Tukey's test.

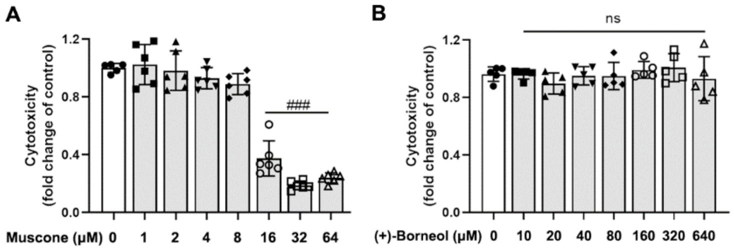

Figure S3 Cytotoxicity of muscone and (+)-borneol on bEnd.3 cells. Cell viability of bEnd.3 cells when treated with the indicated concentrations of muscone (**A**) or (+)-

borneol (**B**) was measured by CCK-8 ($n = 5$). Data are presented as mean ± SD ($n = 5$). $^{###}p < 0.001$ *vs.* untreated group; ns: no significant difference. p values are determined by one-way ANOVA followed by Tukey's test.

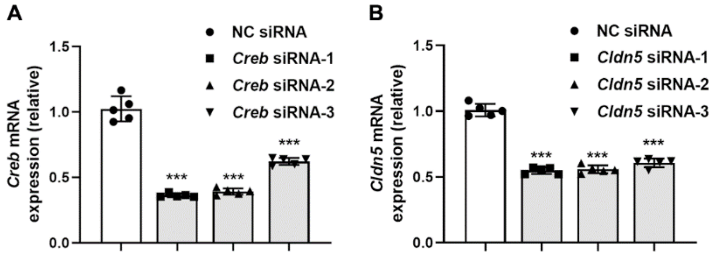

Figure S4 Small interfering RNA (siRNA) screening. bEnd.3 cells were transfected with *Creb* siRNA, *Cldn5* siRNA, or NC siRNA using Lipofectamine 3000 transfection reagent for 48 h. (**A-B**): the gene expressions of *Creb* and *Cldn5* were determined by qRT-PCR ($n = 5$). All data are presented as mean ± SD of five independent experiments. $^{***}p < 0.001$ vs. NC siRNA. p values are determined by one-way ANOVA followed by Tukey's test.

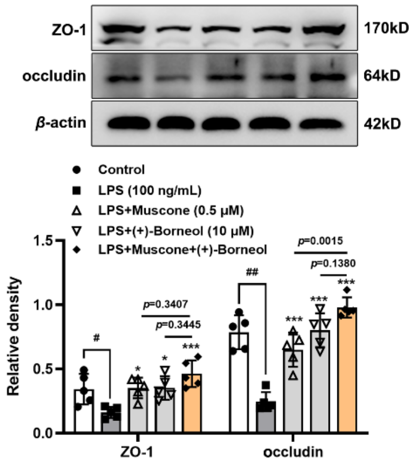

Figure S5 Muscone and (+)-borneol increased ZO-1 and occludin expression. Protein levels of ZO-1 and occludin in LPS-induced bEnd.3 cells treated with muscone and (+)-borneol for 16 h ($n = 5$). Data are presented as mean ± SD ($n = 5$). $^{*}p < 0.05$,

$^{***}p < 0.001$ vs. LPS treatment; $^{\#}p < 0.05$, $^{\#\#}p < 0.01$ vs. indicated treatment. p values are determined by one-way ANOVA followed by Tukey's test.

Table S1. Primer sequences for qRT-PCR

**Primers**		**Sequences**	**Accession number**
Il1β	Forward	5'- TCGCAGCAGCACATCAACAAGAG -3'	AK225002
	Reverse	5'- AGGTCCACGGGAAAGACACAGG -3'	
Cldn5	Forward	5'- TGGTGCTGTGTCTGGTAGGATGG -3'	AK077282
	Reverse	5'- GTCACGATGTTGTGGTCCAGGAAG -3'	
Creb	Forward	5'- CCAGGTCCATGGCGTTATCC -3'	AF448507
	Reverse	5'- ACTGCCCACTGCTAGTTTGG -3'	
Actb	Forward	5'- CTACCTCATGAAGATCCTGACC -3'	AK147787
	Reverse	5'- CACAGCTTCTCTTTGATGTCAC -3'	

Table S2. Primer sequences for ChIP-qPCR

**Primers**		**Sequences**	**Accession number**
Cldn5 promoter (site 1)	Forward	5'- TCTCCCGCCGTCTTCTAAGA-3'	AC083895
	Reverse	5'- TCCCTGCTCCCTTTGTTTCC-3'	
Cldn5 promoter (site 2)	Forward	5'- CTGCTGTCCCAGGATTCCAG-3'	
	Reverse	5'- CAACCACCGCCTACAGTTCT-3'	
